# Supplementary material for: Taste triggers a homeostatic temperature control in hungry flies
Source: eLife. 2024 Dec 2;13:RP94703. doi: 10.7554/eLife.94703 (PMC11611295; doi:10.7554/eLife.94703)
Supplement: Figure 1—source data 1. [file elife-94703-fig1-data1.docx]

Fig. 1D-G and S1

| *w^1118^* | | |
| --- | --- | --- |
| Comparison of Tp between | | p value |
| Fed vs | Starvation | **** |
|  | Refed fly food for 5 min | **** |
|  | Refed fly food for 10 min | ns |
|  | Refed fly food for 30 min | ns |
|  | Refed fly food for 1 hr | ns |
|  | Refed Sucralose for 10 min | *** |
|  | Refed Sucralose for 1 hr | **** |
|  | Refed Glucose for 10 min | ns |
|  | Refed Glucose for 1 hr | ns |
|  | Refed Fructose for 10 min | ns |
|  | Refed Fructose for 1 hr | ns |
| Starvation vs | Refed fly food for 5 min | *** |
|  | Refed fly food for 10 min | **** |
|  | Refed fly food for 30 min | **** |
|  | Refed fly food for 1 hr | **** |
|  | Refed Sucralose for 10 min | * |
|  | Refed Sucralose for 1 hr | * |
|  | Refed Glucose for 10 min | **** |
|  | Refed Glucose for 1 hr | **** |
|  | Refed Fructose for 10 min | **** |
|  | Refed Fructose for 1 hr | **** |

| p value | p<0.0001 |
| --- | --- |
| alpha | 0.05 |
| Multiple test (ANOVA and Tukey’s post hoc test or Kruskal-Wallis test and Dunn’s test) | Tukey test |
| F value (F (DFn, DFd)) | F (12, 75)=16.24 |

Fig. 1H

| *w^1118^*, Cumulative # of licking | | |
| --- | --- | --- |
| Time | Starvation vs Refed Sucralose | Starvation vs Refed Glucose |
| 0-5 | ** | * |
| 5-10 | ** | * |
| 10-15 | ** | * |
| 15-20 | ** | * |
| 20-25 | ** | * |
| 25-30 | ** | ns |

| alpha | 0.05 |
| --- | --- |
| p value |  |
| Time x Refeeding conditions | <0.0001 |
| Time | <0.0001 |
| Refeeding conditions | 0.015 |
| Subject | <0.0001 |
| F (DFn, DFd) |  |
| Time x Refeeding conditions | F (24, 1140) = 3.561 |
| Time | F (1.327, 126.1) = 59.54 |
| Refeeding conditions | F (2, 95) = 4.389 |
| Subject | F (95, 1140) = 31.76 |
