# Supplementary material for: Taste triggers a homeostatic temperature control in hungry flies
Source: eLife. 2024 Dec 2;13:RP94703. doi: 10.7554/eLife.94703 (PMC11611295; doi:10.7554/eLife.94703)
Supplement: Figure 2—source data 1. [file elife-94703-fig2-data1.docx]

Fig. 2

Figs. 2A

| Gr5a[-/-]; Gr64a[-/-] | | |
| --- | --- | --- |
| Comparison of Tp between | | p value |
| Fed vs | Starvation | ** |
|  | Refed fly food for 10 min | ns |
|  | Refed Sucralose for 10 min | *** |
|  | Refed Glucose for 10 min | * |
|  | Refed Glucose for 1 hr | ns |
| Starvation vs | Refed fly food for 10 min | * |
|  | Refed Sucralose for 10 min | ns |
|  | Refed Glucose for 10 min | ns |
|  | Refed Glucose for 1 hr | ns |

| p value | <0.0001 |
| --- | --- |
| alpha | 0.05 |
| Multiple test (ANOVA and Tukey’s post hoc test or Kruskal-Wallis test and Dunn’s test) | Tukey test |
| F value (F (DFn, DFd)) | F (4, 36) = 17.15 |

Fig. 2B

| Gr5a[-/-]; Gr61a[-/-], Gr64a-f[-/-] | | |
| --- | --- | --- |
| Comparison of Tp between | | p value |
| Fed vs | Starvation | **** |
|  | Refed fly food for 10 min | ns |
|  | Refed Sucralose for 10 min | **** |
|  | Refed Glucose for 10 min | *** |
|  | Refed Glucose for 1 hr | * |
| Starvation vs | Refed fly food for 10 min | *** |
|  | Refed Sucralose for 10 min | ns |
|  | Refed Glucose for 10 min | ns |
|  | Refed Glucose for 1 hr | ns |

| p value | P<0.0001 |
| --- | --- |
| alpha | 0.05 |
| Multiple test (ANOVA and Tukey’s post hoc test or Kruskal-Wallis test and Dunn’s test) | Tukey test |
| F value (F (DFn, DFd)) | F (5, 32) = 12.07 |

Fig. 2C

| Gr64f-Gal4>uas-Kir | | |
| --- | --- | --- |
| Comparison of Tp between | | p value |
| Fed vs | Starvation | ** |
|  | Refed fly food for 10 min | ns |
|  | Refed Sucralose for 10 min | * |
|  | Refed Glucose for 10 min | ns |
|  | Refed Glucose for 1 hr | ns |
| Starvation vs | Refed fly food for 10 min | ** |
|  | Refed Sucralose for 10 min | ns |
|  | Refed Glucose for 10 min | ns |
|  | Refed Glucose for 1 hr | ns |

| p value | P<0.0001 |
| --- | --- |
| alpha | 0.05 |
| Multiple test (ANOVA and Tukey’s post hoc test or Kruskal-Wallis test and Dunn’s test) | Tukey test |
| F value (F (DFn, DFd)) | F (6, 58) = 6.277 |

Fig. 2D

| Gr64fGal4/+ | | |
| --- | --- | --- |
| Comparison of Tp between | | p value |
| Fed vs | Starvation | *** |
|  | Refed fly food for 10 min | ns |
|  | Refed Sucralose for 10 min | ns |
|  | Refed Glucose for 10 min | ns |
| Starvation vs | Refed fly food for 10 min | ** |
|  | Refed Sucralose for 10 min | * |
|  | Refed Glucose for 10 min | ** |

| p value | P=0.0001 |
| --- | --- |
| alpha | 0.05 |
| Multiple test (ANOVA and Tukey’s post hoc test or Kruskal-Wallis test and Dunn’s test) | Tukey test |
| F value (F (DFn, DFd)) | F (4, 23) = 9.184 |

Fig. 2E

| uas-Kir/+ | | |
| --- | --- | --- |
| Comparison of Tp between | | p value |
| Fed vs | Starvation | **** |
|  | Refed fly food for 10 min | ns |
|  | Refed Sucralose for 10 min | * |
|  | Refed Glucose for 10 min | ns |
| Starvation vs | Refed fly food for 10 min | **** |
|  | Refed Sucralose for 10 min | ** |
|  | Refed Glucose for 10 min | **** |

| p value | P<0.0001 |
| --- | --- |
| alpha | 0.05 |
| Multiple test (ANOVA and Tukey’s post hoc test or Kruskal-Wallis test and Dunn’s test) | Tukey test |
| F value (F (DFn, DFd)) | F (4, 29) = 20.32 |

Fig. 2G

| Gr64fGal4>uas-CsChrimson | | |
| --- | --- | --- |
| Comparison of Tp between | | p value |
| Fed vs | Water + Red light | **** |
|  | ATR + Red light | * |
| Water + Red light vs | ATR + Red light | * |

| p value | P<0.0001 |
| --- | --- |
| alpha | 0.05 |
| Multiple test (ANOVA and Tukey’s post hoc test or Kruskal-Wallis test and Dunn’s test) | Tukey test |
| F value (F (DFn, DFd)) | F (2, 23) = 14.35 |

Fig. 2H

| Gr5aGal4>uas-CsChrimson | | |
| --- | --- | --- |
| Comparison of Tp between | | p value |
| Fed vs | Water + Red light | * |
|  | ATR + Red light | * |
| Water + Red light vs | ATR + Red light | ns |

| p value | P=0.0150 |
| --- | --- |
| alpha | 0.05 |
| Multiple test (ANOVA and Tukey’s post hoc test or Kruskal-Wallis test and Dunn’s test) | Tukey test |
| F value (F (DFn, DFd)) | F (2, 12) = 6.084 |

Fig. 2I

| Gr64aGal4>uas-CsChrimson | | |
| --- | --- | --- |
| Comparison of Tp between | | p value |
| Fed vs | Water + Red light | **** |
|  | ATR + Red light | **** |
| Water + Red light vs | ATR + Red light | ns |

| p value | P<0.0001 |
| --- | --- |
| alpha | 0.05 |
| Multiple test (ANOVA and Tukey’s post hoc test or Kruskal-Wallis test and Dunn’s test) | Tukey test |
| F value (F (DFn, DFd)) | F (2, 23) = 21.72 |

Fig. 2J

| uas-CsChrimson/+ | | |
| --- | --- | --- |
| Comparison of Tp between | | p value |
| Fed vs | Water + Red light | *** |
|  | ATR + Red light | * |
| Water + Red light vs | ATR + Red light | ns |

| p value | P=0.0010 |
| --- | --- |
| alpha | 0.05 |
| Multiple test (ANOVA and Tukey’s post hoc test or Kruskal-Wallis test and Dunn’s test) | Tukey test |
| F value (F (DFn, DFd)) | F (2, 13) = 12.25 |
