# Supplementary material for: Taste triggers a homeostatic temperature control in hungry flies
Source: eLife. 2024 Dec 2;13:RP94703. doi: 10.7554/eLife.94703 (PMC11611295; doi:10.7554/eLife.94703)
Supplement: Figure 3—source data 1. [file elife-94703-fig3-data1.docx]

Fig. 3

Figs. 3A

| TrpA1[SH]Gal4>uas-Kir | | |
| --- | --- | --- |
| Comparison of Tp between | | p value |
| Fed vs | Starvation | ** |
|  | Refed fly food for 10 min | ns |
|  | Refed Sucralose for 10 min | **** |
|  | Refed Glucose for 10 min | ns |
|  | Refed Glucose for 1 hr | ns |
| Starvation vs | Refed fly food for 10 min | * |
|  | Refed Sucralose for 10 min | ns |
|  | Refed Glucose for 10 min | ns |
|  | Refed Glucose for 1 hr | *** |

| p value | P<0.0001 |
| --- | --- |
| alpha | 0.05 |
| Multiple test (ANOVA and Tukey’s post hoc test or Kruskal-Wallis test and Dunn’s test) | Tukey test |
| F value (F (DFn, DFd)) | F (5, 42) = 10.02 |

Figs. 3B

| R11F02Gal4>uas-Kir | | |
| --- | --- | --- |
| Comparison of Tp between | | p value |
| Fed vs | Starvation | * |
|  | Refed fly food for 10 min | ns |
|  | Refed Sucralose for 10 min | ns |
|  | Refed Glucose for 10 min | ns |
|  | Refed Glucose for 1 hr | ns |
| Starvation vs | Refed fly food for 10 min | * |
|  | Refed Sucralose for 10 min | ns |
|  | Refed Glucose for 10 min | ns |
|  | Refed Glucose for 1 hr | ns |

| p value | 0.0016 |
| --- | --- |
| alpha | 0.05 |
| Multiple test (ANOVA and Tukey’s post hoc test or Kruskal-Wallis test and Dunn’s test) | Dunn's test |
| F value (F (DFn, DFd)) |  |

Figs. 3C

| TrpA1[SH]Gal4/+ | | |
| --- | --- | --- |
| Comparison of Tp between | | p value |
| Fed vs | Starvation | ** |
|  | Refed fly food for 10 min | ns |
|  | Refed Sucralose for 10 min | ns |
|  | Refed Glucose for 10 min | ns |
| Starvation vs | Refed fly food for 10 min | ** |
|  | Refed Sucralose for 10 min | * |
|  | Refed Glucose for 10 min | *** |

| p value | P=0.0003 |
| --- | --- |
| alpha | 0.05 |
| Multiple test (ANOVA and Tukey’s post hoc test or Kruskal-Wallis test and Dunn’s test) | Tukey test |
| F value (F (DFn, DFd)) | F (4, 31) = 7.442 |

Figs. 3D

| R11F02Gal4/+ | | |
| --- | --- | --- |
| Comparison of Tp between | | p value |
| Fed vs | Starvation | *** |
|  | Refed fly food for 10 min | ns |
|  | Refed Sucralose for 10 min | ns |
|  | Refed Glucose for 10 min | ns |
| Starvation vs | Refed fly food for 10 min | ** |
|  | Refed Sucralose for 10 min | * |
|  | Refed Glucose for 10 min | *** |

| p value | P<0.0001 |
| --- | --- |
| alpha | 0.05 |
| Multiple test (ANOVA and Tukey’s post hoc test or Kruskal-Wallis test and Dunn’s test) | Tukey test |
| F value (F (DFn, DFd)) | F (4, 40) = 8.637 |

Fig. 3F

| TrpA1[SH]Gal4>uas-CsChrimson | | |
| --- | --- | --- |
| Comparison of Tp between | | p value |
| Water + Red light vs | ATR + Red light | * |

| p value | P=0.0186 |
| --- | --- |
| alpha | 0.05 |
| t-test or Kolmogorov-Smirnov test | Kolmogorov-Smirnov test |

Fig. 3G

| R11F02Gal4>uas-CsChrimson, tubGal80[ts] | | |
| --- | --- | --- |
| Comparison of Tp between | | p value |
| Water + Red light vs | ATR + Red light | * |

| p value | P=0.0192 |
| --- | --- |
| alpha | 0.05 |
| t-test or Kolmogorov-Smirnov test | Unpaired t-test |

Fig. 3H

| *Orco^1^* | | |
| --- | --- | --- |
| Comparison of Tp between | | p value |
| Fed vs | Starvation | **** |
|  | Refed fly food for 10 min | *** |
|  | Refed Sucralose for 10 min | **** |
|  | Refed Glucose for 10 min | *** |
|  | Refed Glucose for 1 hr | ns |
| Starvation vs | Refed fly food for 10 min | * |
|  | Refed Sucralose for 10 min | ns |
|  | Refed Glucose for 10 min | * |
|  | Refed Glucose for 1 hr | **** |

| p value | P<0.0001 |
| --- | --- |
| alpha | 0.05 |
| Multiple test (One-way Anova and Tukey's test or Kraskal-Wallis test and Dunn’s test) | Tuckey's test |
| F value (F (DFn, DFd)) | F (5, 49) = 18.90 |
