# Supplementary material for: Taste triggers a homeostatic temperature control in hungry flies
Source: eLife. 2024 Dec 2;13:RP94703. doi: 10.7554/eLife.94703 (PMC11611295; doi:10.7554/eLife.94703)
Supplement: Figure 3—figure supplement 1—source data 1. [file elife-94703-fig3-figsupp1-data1.docx]

Fig. S3

| w[1118] | | |
| --- | --- | --- |
| Comparison of Tp between | | p value |
| Fed vs | Starvation for 1 ON (STV1ON) | **** |
|  | STV1ON+Refed Sucralose for 10 min | *** |
|  | STV1ON+Refed Glucose for 10 min | ns |
| STV1ON vs | STV1ON+Refed Sucralose for 10 min | ** |
|  | STV1ON+Refed Glucose for 10 min | **** |
| Fed vs | Starvation for 1.5 ON (STV1.5ON) | **** |
|  | STV1.5ON+Refed Sucralose for 10 min | ** |
|  | STV1.5ON+Refed Glucose for 10 min | ns |
| STV1.5ON vs | STV1.5ON+Refed Sucralose for 10 min | ** |
|  | STV1.5ON+Refed Glucose for 10 min | **** |

| p value | P<0.0001 |
| --- | --- |
| alpha | 0.05 |
| Multiple test (ANOVA and Tukey’s post hoc test or Kruskal-Wallis test and Dunn’s test) | Tukey test |
| F value (F (DFn, DFd)) | F (6, 42)=23.02 |
