# Supplementary material for: Taste triggers a homeostatic temperature control in hungry flies
Source: eLife. 2024 Dec 2;13:RP94703. doi: 10.7554/eLife.94703 (PMC11611295; doi:10.7554/eLife.94703)
Supplement: Figure 4—source data 1. [file elife-94703-fig4-data1.docx]

Fig. 4

Figs. 4A

| NPF[-/-] | | |
| --- | --- | --- |
| Comparison of Tp between | | p value |
| Fed vs | Starvation | **** |
|  | Refed fly food for 10 min | *** |
|  | Refed Sucralose for 10 min | **** |
|  | Refed Glucose for 10 min | **** |
|  | Refed Glucose for 1 hr | ** |
| Starvation vs | Refed fly food for 10 min | * |
|  | Refed Sucralose for 10 min | ns |
|  | Refed Glucose for 10 min | ns |
|  | Refed Glucose for 1 hr | *** |

| p value | P<0.0001 |
| --- | --- |
| alpha | 0.05 |
| Multiple test (ANOVA and Tukey’s post hoc test or Kruskal-Wallis test and Dunn’s test) | Tukey test |
| F value (F (DFn, DFd)) | F (5, 44) = 14.01 |

Figs. 4B

| sNPF hypo | | |
| --- | --- | --- |
| Comparison of Tp between | | p value |
| Fed vs | Starvation | **** |
|  | Refed fly food for 10 min | * |
|  | Refed Sucralose for 10 min | **** |
|  | Refed Glucose for 10 min | *** |
|  | Refed Glucose for 1 hr | *** |
| Starvation vs | Refed fly food for 10 min | ** |
|  | Refed Sucralose for 10 min | ns |
|  | Refed Glucose for 10 min | ** |
|  | Refed Glucose for 1 hr | ** |

| p value | P<0.0001 |
| --- | --- |
| alpha | 0.05 |
| Multiple test (ANOVA and Tukey’s post hoc test or Kruskal-Wallis test and Dunn’s test) | Tuckey test |
| F value (F (DFn, DFd)) | F (5, 39) = 18.35 |

Figs. 4C

| Dh44Gal4/+ | | |
| --- | --- | --- |
| Comparison of Tp between | | p value |
| Fed vs | Starvation | **** |
|  | Refed fly food for 10 min | ns |
|  | Refed Sucralose for 10 min | ** |
|  | Refed Glucose for 10 min | ns |
| Starvation vs | Refed fly food for 10 min | **** |
|  | Refed Sucralose for 10 min | **** |
|  | Refed Glucose for 10 min | **** |

| p value | P<0.0001 |
| --- | --- |
| alpha | 0.05 |
| Multiple test (ANOVA and Tukey’s post hoc test or Kruskal-Wallis test and Dunn’s test) | Tukey test |
| F value (F (DFn, DFd)) | F (4, 25) = 27.23 |

Figs. 4D

| Dh44Gal4>uas-Kir | | |
| --- | --- | --- |
| Comparison of Tp between | | p value |
| Fed vs | Starvation | **** |
|  | Refed fly food for 10 min | ns |
|  | Refed Sucralose for 10 min | **** |
|  | Refed Glucose for 10 min | * |
| Starvation vs | Refed fly food for 10 min | **** |
|  | Refed Sucralose for 10 min | ns |
|  | Refed Glucose for 10 min | *** |

| p value | P<0.0001 |
| --- | --- |
| alpha | 0.05 |
| Multiple test (ANOVA and Tukey’s post hoc test or Kruskal-Wallis test and Dunn’s test) | Tukey test |
| F value (F (DFn, DFd)) | F (4, 38) = 21.93 |

Figs. 4E

| AkhGal4/+ | | |
| --- | --- | --- |
| Comparison of Tp between | | p value |
| Fed vs | Starvation | **** |
|  | Refed fly food for 10 min | ns |
|  | Refed Sucralose for 10 min | ns |
|  | Refed Glucose for 10 min | ns |
| Starvation vs | Refed fly food for 10 min | *** |
|  | Refed Sucralose for 10 min | *** |
|  | Refed Glucose for 10 min | *** |

| p value | P<0.0001 |
| --- | --- |
| alpha | 0.05 |
| Multiple test (ANOVA and Tukey’s post hoc test or Kruskal-Wallis test and Dunn’s test) | Tukey test |
| F value (F (DFn, DFd)) | F (4, 30) = 14.32 |

Figs. 4F

| AkhGal4>uas-Kir | | |
| --- | --- | --- |
| Comparison of Tp between | | p value |
| Fed vs | Starvation | **** |
|  | Refed fly food for 10 min | ns |
|  | Refed Sucralose for 10 min | *** |
|  | Refed Glucose for 10 min | ns |
|  | Refed Glucose for 1 hr | ns |
| Starvation vs | Refed fly food for 10 min | **** |
|  | Refed Sucralose for 10 min | ns |
|  | Refed Glucose for 10 min | **** |
|  | Refed Glucose for 1 hr | **** |

| p value | P<0.0001 |
| --- | --- |
| alpha | 0.05 |
| Multiple test (ANOVA and Tukey’s post hoc test or Kruskal-Wallis test and Dunn’s test) | Tukey test |
| F value (F (DFn, DFd)) | F (5, 35) = 16.47 |

Figs. 4G

| ilp6 LOF | | |
| --- | --- | --- |
| Comparison of Tp between | | p value |
| Fed vs | Starvation | **** |
|  | Refed fly food for 10 min | ns |
|  | Refed Sucralose for 10 min | **** |
|  | Refed Glucose for 10 min | ns |
|  | Refed Glucose for 1 hr | ns |
| Starvation vs | Refed fly food for 10 min | **** |
|  | Refed Sucralose for 10 min | ns |
|  | Refed Glucose for 10 min | **** |
|  | Refed Glucose for 1 hr | **** |

| p value | P<0.0001 |
| --- | --- |
| alpha | 0.05 |
| Multiple test (ANOVA and Tukey’s post hoc test or Kruskal-Wallis test and Dunn’s test) | Tukey test |
| F value (F (DFn, DFd)) | F (5, 48) = 23.13 |

Figs. 4H

| Upd3Δ | | |
| --- | --- | --- |
| Comparison of Tp between | | p value |
| Fed vs | Starvation | **** |
|  | Refed fly food for 10 min | ns |
|  | Refed Sucralose for 10 min | **** |
|  | Refed Glucose for 10 min | ns |
|  | Refed Glucose for 1 hr | ns |
| Starvation vs | Refed fly food for 10 min | ns |
|  | Refed Sucralose for 10 min | ns |
|  | Refed Glucose for 10 min | * |
|  | Refed Glucose for 1 hr | ** |

| p value | P<0.0001 |
| --- | --- |
| alpha | 0.05 |
| Multiple test (ANOVA and Tukey’s post hoc test or Kruskal-Wallis test and Dunn’s test) | Tukey test |
| F value (F (DFn, DFd)) | F (5, 37) = 14.92 |

Figs. 4I

| Upd2Δ | | |
| --- | --- | --- |
| Comparison of Tp between | | p value |
| Fed vs | Starvation | ** |
|  | Refed fly food for 10 min | ns |
|  | Refed Sucralose for 10 min | **** |
|  | Refed Glucose for 10 min | ns |
|  | Refed Glucose for 1 hr | ns |
| Starvation vs | Refed fly food for 10 min | ns |
|  | Refed Sucralose for 10 min | ns |
|  | Refed Glucose for 10 min | ns |
|  | Refed Glucose for 1 hr | ns |

| p value | P=0.0002 |
| --- | --- |
| alpha | 0.05 |
| Multiple test (ANOVA and Tukey’s post hoc test or Kruskal-Wallis test and Dunn’s test) | Tukey test |
| F value (F (DFn, DFd)) | F (5, 43) = 6.356 |
