# Supplementary material for: Taste triggers a homeostatic temperature control in hungry flies
Source: eLife. 2024 Dec 2;13:RP94703. doi: 10.7554/eLife.94703 (PMC11611295; doi:10.7554/eLife.94703)
Supplement: Figure 4—figure supplement 1—source data 1. [file elife-94703-fig4-figsupp1-data1.docx]

Fig. S4A

| *yw, Cumulative # of licking* | | |
| --- | --- | --- |
| Time | Starvation vs Refed Sucralose | Starvation vs Refed Glucose |
| 0-5 | ns | ns |
| 5-10 | ns | ns |
| 10-15 | ns | ns |
| 15-20 | ns | ns |
| 20-25 | ns | ns |
| 25-30 | ns | ns |

| alpha | 0.05 |
| --- | --- |
| p value |  |
| Time x Refeeding conditions) | 0.9999 |
| Time | <0.0001 |
| Refeeding conditions | 0.8321 |
| Subject | <0.0001 |
| F (DFn, DFd) |  |
| Time x Refeeding conditions | F (24, 1704) = 0.2587 |
| Time | F (1.189, 168.8) = 102.8 |
| Refeeding conditions | F (2, 142) = 0.1841 |
| Subject | F (142, 1704) = 27.38 |

Fig. S4B

| *per^01^, Cumulative # of licking* | | |
| --- | --- | --- |
| Time | Starvation vs Refed Sucralose | Starvation vs Refed Glucose |
| 0-5 | *** | ns |
| 5-10 | ** | ns |
| 10-15 | ** | ns |
| 15-20 | *** | ns |
| 20-25 | *** | ns |
| 25-30 | *** | ns |

| alpha | 0.05 |
| --- | --- |
| p value |  |
| Time x Refeeding conditions) | <0.0001 |
| Time | <0.0001 |
| Refeeding conditions | 0.0018 |
| Subject | <0.0001 |
| F (DFn, DFd) |  |
| Time x Refeeding conditions | F (24, 1476) = 6.720 |
| Time | F (1.137, 139.9) = 73.68 |
| Refeeding conditions | F (2, 123) = 6.686 |
| Subject | F (123, 1476) = 32.81 |

Fig. S4C

| *tim^01^, Cumulative # of licking* | | |
| --- | --- | --- |
| Time | Starvation vs Refed Sucralose | Starvation vs Refed Glucose |
| 0-5 | ns | * |
| 5-10 | ns | ** |
| 10-15 | ns | * |
| 15-20 | ns | * |
| 20-25 | ns | * |
| 25-30 | ns | ns |

| alpha | 0.05 |
| --- | --- |
| p value |  |
| Time x Refeeding conditions) | 0.5253 |
| Time | <0.0001 |
| Refeeding conditions | 0.211 |
| Subject | <0.0001 |
| F (DFn, DFd) |  |
| Time x Refeeding conditions | F (24, 1668) = 0.9547 |
| Time | F (1.182, 164.2) = 190.0 |
| Refeeding conditions | F (2, 139) = 1.574 |
| Subject | F (139, 1668) = 27.06 |
