# Supplementary material for: Taste triggers a homeostatic temperature control in hungry flies
Source: eLife. 2024 Dec 2;13:RP94703. doi: 10.7554/eLife.94703 (PMC11611295; doi:10.7554/eLife.94703)
Supplement: Figure 5—source data 1. [file elife-94703-fig5-data1.docx]

Fig. 5

Fig. 5A

w[1118], LD

| w[1118], LD, ZT1-3 | | |
| --- | --- | --- |
| Comparison of Tp between | | p value |
| Fed vs | Starvation | *** |
|  | Refed Sucralose for 10 min | ** |
|  | Refed Glucose for 10 min | NS |
|  | Refed Glucose for 1 hr | NS |
| Starvation vs | Refed Sucralose for 10 min | NS |
|  | Refed Glucose for 10 min | **** |
|  | Refed Glucose for 1 hr | **** |

| p value | P<0.0001 |
| --- | --- |
| alpha | 0.05 |
| Multiple test (ANOVA and Tukey’s post hoc test or Kruskal-Wallis test and Dunn’s test) | Tukey test |
| F value (F (DFn, DFd)) | F (4, 26) = 14.24 |

| w[1118], LD, ZT4-6 | | |
| --- | --- | --- |
| Comparison of Tp between | | p value |
| Fed vs | Starvation | **** |
|  | Refed Sucralose for 10 min | NS |
|  | Refed Glucose for 10 min | NS |
|  | Refed Glucose for 1 hr | NS |
| Starvation vs | Refed Sucralose for 10 min | ** |
|  | Refed Glucose for 10 min | *** |
|  | Refed Glucose for 1 hr | *** |

| p value | P<0.0001 |
| --- | --- |
| alpha | 0.05 |
| Multiple test (ANOVA and Tukey’s post hoc test or Kruskal-Wallis test and Dunn’s test) | Tukey test |
| F value (F (DFn, DFd)) | F (4, 36) = 9.693 |

| w[1118], LD, ZT7-9 | | |
| --- | --- | --- |
| Comparison of Tp between | | p value |
| Fed vs | Starvation | ** |
|  | Refed Sucralose for 10 min | NS |
|  | Refed Glucose for 10 min | NS |
|  | Refed Glucose for 1 hr | NS |
| Starvation vs | Refed Sucralose for 10 min | NS |
|  | Refed Glucose for 10 min | NS |
|  | Refed Glucose for 1 hr | * |

| p value | P=0.0054 |
| --- | --- |
| alpha | 0.05 |
| Multiple test (ANOVA and Tukey’s post hoc test or Kruskal-Wallis test and Dunn’s test) | Tukey test |
| F value (F (DFn, DFd)) | F (4, 25) = 4.768 |

| w[1118], LD, ZT10-12 | | |
| --- | --- | --- |
| Comparison of Tp between | | p value |
| Fed vs | Starvation | **** |
|  | Refed Sucralose for 10 min | * |
|  | Refed Glucose for 10 min | NS |
|  | Refed Glucose for 1 hr | NS |
| Starvation vs | Refed Sucralose for 10 min | **** |
|  | Refed Glucose for 10 min | **** |
|  | Refed Glucose for 1 hr | **** |

| p value | P=0.0054 |
| --- | --- |
| alpha | 0.05 |
| Multiple test (ANOVA and Tukey’s post hoc test or Kruskal-Wallis test and Dunn’s test) | Tukey test |
| F value (F (DFn, DFd)) | F (4, 25) = 4.768 |

Fig. 5B

y[1]w[1], LD

| y[1]w[1], LD, ZT1-3 | | |
| --- | --- | --- |
| Comparison of Tp between | | p value |
| Fed vs | Starvation | **** |
|  | Refed Sucralose for 10 min | ns |
|  | Refed Glucose for 10 min | ns |
| Starvation vs | Refed Sucralose for 10 min | *** |
|  | Refed Glucose for 10 min | ** |

| p value | P<0.0001 |
| --- | --- |
| alpha | 0.05 |
| Multiple test (ANOVA and Tukey’s post hoc test or Kruskal-Wallis test and Dunn’s test) | Tukey test |
| F value (F (DFn, DFd)) | F (3, 32) = 12.04 |

| y[1]w[1], LD, ZT4-6 | | |
| --- | --- | --- |
| Comparison of Tp between | | p value |
| Fed vs | Starvation | ** |
|  | Refed Sucralose for 10 min | ns |
|  | Refed Glucose for 10 min | ns |
| Starvation vs | Refed Sucralose for 10 min | * |
|  | Refed Glucose for 10 min | ** |

| p value | P<0.0001 |
| --- | --- |
| alpha | 0.05 |
| Multiple test (ANOVA and Tukey’s post hoc test or Kruskal-Wallis test and Dunn’s test) | Dunn's test |
| F value (F (DFn, DFd)) |  |

| y[1]w[1], LD, ZT7-9 | | |
| --- | --- | --- |
| Comparison of Tp between | | p value |
| Fed vs | Starvation | **** |
|  | Refed Sucralose for 10 min | NS |
|  | Refed Glucose for 10 min | NS |
| Starvation vs | Refed Sucralose for 10 min | *** |
|  | Refed Glucose for 10 min | **** |

| p value | P<0.0001 |
| --- | --- |
| alpha | 0.05 |
| Multiple test (ANOVA and Tukey’s post hoc test or Kruskal-Wallis test and Dunn’s test) | Tukey test |
| F value (F (DFn, DFd)) | F (3, 32) = 23.95 |

| y[1]w[1], LD, ZT10-12 | | |
| --- | --- | --- |
| Comparison of Tp between | | p value |
| Fed vs | Starvation | **** |
|  | Refed Sucralose for 10 min | NS |
|  | Refed Glucose for 10 min | NS |
| Starvation vs | Refed Sucralose for 10 min | **** |
|  | Refed Glucose for 10 min | **** |

| p value | P=0.0001 |
| --- | --- |
| alpha | 0.05 |
| Multiple test (ANOVA and Tukey’s post hoc test or Kruskal-Wallis test and Dunn’s test) | Dunn's test |
| F value (F (DFn, DFd)) |  |

Fig. 5C

per[01], LD

| per[01], LD, ZT1-3 | | |
| --- | --- | --- |
| Comparison of Tp between | | p value |
| Fed vs | Starvation | ** |
|  | Refed Sucralose for 10 min | **** |
|  | Refed Glucose for 10 min | ns |
| Starvation vs | Refed Sucralose for 10 min | ns |
|  | Refed Glucose for 10 min | **** |

| p value | P<0.0001 |
| --- | --- |
| alpha | 0.05 |
| Multiple test (ANOVA and Tukey’s post hoc test or Kruskal-Wallis test and Dunn’s test) | Tukey test |
| F value (F (DFn, DFd)) | F (3, 23) = 28.56 |

| per[01], LD, ZT4-6 | | |
| --- | --- | --- |
| Comparison of Tp between | | p value |
| Fed vs | Starvation | * |
|  | Refed Sucralose for 10 min | ** |
|  | Refed Glucose for 10 min | ns |
| Starvation vs | Refed Sucralose for 10 min | ns |
|  | Refed Glucose for 10 min | ** |

| p value | P=0.0003 |
| --- | --- |
| alpha | 0.05 |
| Multiple test (ANOVA and Tukey’s post hoc test or Kruskal-Wallis test and Dunn’s test) | Tukey test |
| F value (F (DFn, DFd)) | F (3, 22) = 9.425 |

| per[01], LD, ZT7-9 | | |
| --- | --- | --- |
| Comparison of Tp between | | p value |
| Fed vs | Starvation | ** |
|  | Refed Sucralose for 10 min | * |
|  | Refed Glucose for 10 min | ns |
| Starvation vs | Refed Sucralose for 10 min | ns |
|  | Refed Glucose for 10 min | * |

| p value | P=0.0014 |
| --- | --- |
| alpha | 0.05 |
| Multiple test (ANOVA and Tukey’s post hoc test or Kruskal-Wallis test and Dunn’s test) | Dunn's test |
| F value (F (DFn, DFd)) |  |

| per[01], LD, ZT10-12 | | |
| --- | --- | --- |
| Comparison of Tp between | | p value |
| Fed vs | Starvation | * |
|  | Refed Sucralose for 10 min | *** |
|  | Refed Glucose for 10 min | ns |
| Starvation vs | Refed Sucralose for 10 min | ns |
|  | Refed Glucose for 10 min | * |

| p value | P<0.0001 |
| --- | --- |
| alpha | 0.05 |
| Multiple test (ANOVA and Tukey’s post hoc test or Kruskal-Wallis test and Dunn’s test) | Tukey test |
| F value (F (DFn, DFd)) | F (3, 17) = 13.36 |

Fig. 5D

tim[01], LD

| tim[01], LD, ZT1-3 | | |
| --- | --- | --- |
| Comparison of Tp between | | p value |
| Fed vs | Starvation | **** |
|  | Refed Sucralose for 10 min | **** |
|  | Refed Glucose for 10 min | ns |
| Starvation vs | Refed Sucralose for 10 min | ns |
|  | Refed Glucose for 10 min | **** |

| p value | P<0.0001 |
| --- | --- |
| alpha | 0.05 |
| Multiple test (ANOVA and Tukey’s post hoc test or Kruskal-Wallis test and Dunn’s test) | Tukey test |
| F value (F (DFn, DFd)) | F (3, 18) = 34.89 |

| tim[01], LD, ZT4-6 | | |
| --- | --- | --- |
| Comparison of Tp between | | p value |
| Fed vs | Starvation | **** |
|  | Refed Sucralose for 10 min | **** |
|  | Refed Glucose for 10 min | ns |
| Starvation vs | Refed Sucralose for 10 min | ns |
|  | Refed Glucose for 10 min | **** |

| p value | P<0.0001 |
| --- | --- |
| alpha | 0.05 |
| Multiple test (ANOVA and Tukey’s post hoc test or Kruskal-Wallis test and Dunn’s test) | Tukey test |
| F value (F (DFn, DFd)) | F (3, 32) = 44.40 |

| tim[01], LD, ZT7-9 | | |
| --- | --- | --- |
| Comparison of Tp between | | p value |
| Fed vs | Starvation | **** |
|  | Refed Sucralose for 10 min | **** |
|  | Refed Glucose for 10 min | ns |
| Starvation vs | Refed Sucralose for 10 min | ns |
|  | Refed Glucose for 10 min | **** |

| p value | P<0.0001 |
| --- | --- |
| alpha | 0.05 |
| Multiple test (ANOVA and Tukey’s post hoc test or Kruskal-Wallis test and Dunn’s test) | Tukey test |
| F value (F (DFn, DFd)) | F (3, 39) = 28.77 |

| tim[01], LD, ZT10-12 | | |
| --- | --- | --- |
| Comparison of Tp between | | p value |
| Fed vs | Starvation | *** |
|  | Refed Sucralose for 10 min | ** |
|  | Refed Glucose for 10 min | ns |
| Starvation vs | Refed Sucralose for 10 min | ns |
|  | Refed Glucose for 10 min | * |

| p value | P=0.0002 |
| --- | --- |
| alpha | 0.05 |
| Multiple test (ANOVA and Tukey’s post hoc test or Kruskal-Wallis test and Dunn’s test) | Dunn's test |
| F value (F (DFn, DFd)) |  |
